# TIMELESS regulates sphingolipid metabolism and tumor cell growth through Sp1/ACER2/S1P axis in ER-positive breast cancer

**DOI:** 10.1038/s41419-020-03106-4

**Published:** 2020-10-22

**Authors:** Shan Zhang, Peiqi Huang, Huijuan Dai, Qing Li, Lipeng Hu, Jing Peng, Shuheng Jiang, Yaqian Xu, Ziping Wu, Huizhen Nie, Zhigang Zhang, Wenjin Yin, Xueli Zhang, Jinsong Lu

**Affiliations:** 1 Department of Breast Surgery, Renji Hospital, School of Medicine, Shanghai Jiaotong University, No. 1630 Dongfang Road, Shanghai, 200127 China; 2 State Key Laboratory of Oncogenes and Related Genes, Shanghai Cancer Institute, Renji Hospital, School of Medicine, Shanghai Jiaotong University, Shanghai, 200240 China

**Keywords:** Breast cancer, Cancer metabolism

## Abstract

Breast cancer is one of the most common female malignant cancers. Biorhythm disorder largely increases the risk of breast cancer. We aimed to investigate the biological functions and molecular mechanisms of circadian gene TIMELESS circadian regulator (TIM) in estrogen receptor (ER)-positive breast cancer and provide a new therapeutic target for breast cancer patients. Here, we explored that the expression of TIM was elevated in breast cancer, and high expression of TIM in cancer tissues was associated with poor prognosis, especially in the ER-positive breast cancer patients. In addition, we found that TIM promoted cell proliferation and enhanced mitochondrial respiration. TIM interacted with specificity protein 1 (Sp1) which contributes to upregulate the expression of alkaline ceramidase 2 (ACER2). Moreover, ACER2 is responsible for TIM-mediated promotive effects of cell growth and mitochondrial respiration. Collectively, our research unveiled a novel function of TIM in sphingolipid metabolism through interaction with Sp1. It provides a new theoretical explanation for the pathogenesis of breast cancer, and targeting TIM may serve as a potential therapeutic target for ER-positive breast cancer.

## Introduction

Breast cancer is one of the most common female malignant cancers. According to the reports from the American Cancer Society, there are more than 3.8 million women with a history of invasive breast cancer in the United States, and 276,480 women are newly diagnosed in 2019^1,2^. Estrogen receptor (ER)-positive breast cancer, which is the most common type, accounts for 65–75% of breast cancer^3,4^. The incidence of breast cancer remains very high and the causes of breast cancer is not clearly known yet. The meta-analysis found that ‘ever exposed to night work’ was highly associated with breast cancer. Further meta-analysis of the dose-response relationship showed that for every 5 years of night shift work, the risk of breast cancer increased by 3% in female^5^. Basic studies have demonstrated that dim light can destroy the circadian rhythm through suppressing melatonin signaling pathways, which promotes the growth of breast cancer via metabolism-related signaling^6–8^. These findings suggest that biorhythm disorders are closely associated with the incidence and progression of breast cancer.

Organisms face environmental and periodic changes. To adapt to these changes, organisms generate internal time to maintain physiological processes and the biorhythm is the external manifestation of the internal time of the organism^9–11^. At the cellular level, the biological rhythm is regulated by transcription and translation feedback loops (TTFLs). After the body receives light, food and other external factors to provide time clues, cells regulate the expression of circadian clock genes to participate in a series of physiological circadian rhythm processes^12^. As one of the basic biological behaviors of organisms, the disorder of biological rhythm is related to inflammation, endocrine, and tumor, etc.^13^.

TIM, as a circadian clock gene, has been found to be highly expressed and predictive of poor prognosis in various cancers including lung cancer and breast cancer^14,15^. Accumulated studies have shown the important roles of TIM in modulating DNA damage response, replication stress, and tumor growth. TIM can form a complex with PARP1 involved in the DNA damage repair and then protect cancer cells from apoptosis^16^. Elevated TIM and Claspin improve tolerance of replication stress by protecting replication forks, which is beneficial to tumor growth in different kinds of cancer such as colon cancer and breast cancer^17^. However, the function and the underlying mechanism of TIM in breast cancer remain undefined.

In this study, we found that TIM was significantly upregulated in ER-positive breast cancer and closely correlated with poor prognosis. In vitro and in vivo studies revealed that TIM facilitated the breast cancer cell growth. We further demonstrated that TIM increased the synthesis of S1P via upregulating the expression of ACER2 and improved the mitochondrial respiration. Further mechanistic studies showed that the interaction between TIM and Sp1 regulated the expression of ACER2. Thus, TIM might be served as a potential biomarker and target for the diagnosis and the treatment of breast cancer.

## Materials and methods

### Human sample

A cohort of 76 frozen human breast cancer biopsy specimens came from two neoadjuvant clinical trials, which registered as SHPD001 (NCT02199418) and SHPD002 (NCT02221999) in ClinicalTrials.gov. The SHPD001 and SHPD002 trials were approved by the Independent Ethical Committee of Renji Hospital, School of Medicine, Shanghai JiaoTong University. Before enrollment, all patients signed written informed consent. The specimens were collected from 2016 to 2018. All patients were histopathologically diagnosed with invasive breast cancer. Before neoadjuvant chemotherapy, none of the patients had received any anti-tumor therapy.

### Cell lines and reagents

All the human breast cancer cell lines MCF7, T47D, ZR75-30, Bcap37, Sk-BR-3, MDA-MB-231, MDA-MB-468, and normal mammary epithelial cell line HBL100 were obtained from the American Type Culture Collection (ATCC). The cultured medium followed the ATCC protocols and supplemented with 10% (volume/volume) fetal bovine serum and 1% antibiotics (penicillin and streptomycin) at 37 °C in a humidified incubator under 5% CO_2_ condition.

The reagents used were C18-S1P (73914, Sigma-Aldrich, St. Louis, MO, USA), C17-S1P (LM2144, Avanti, St. Louis, MO, USA), Doxycycline (D9891, Sigma-Aldrich, St. Louis, MO, USA), anti-rabbit lgG (AB0101, Abways, Shanghai, China), and anti-mouse IgG light chain (A25012, Abbkine, CA, USA).

### Cell transfection

To knock down gene expression, small interfering RNA (siRNA) was transfected to the cells with Lipofectamine RNAiMAX (Invitrogen). The sequences of the siRNA used were as follows:

siTIM-1, 5′-GCUAGAGAUUGUCUCCCUUTT-3′;

siTIM-2, 5′-CCAAAUACAUCCUGGGCAATT-3′;

siSp1-1, 5′-GCAACAUCAUUGCUGCUAUTT-3′;

siSp1-2, 5′-CCAUUAACCUCAGUGCAUUTT-3′.

Negative control was scrambled siRNA targeting no known gene sequence. The transfection steps were performed according to the manufacture’s protocols. Lipofectamine 2000 (11668-019, Invitrogen) was implemented in the TIM, ACER2 and Alkaline Ceramidase 3 (ACER3) overexpressing plasmids and its vector plasmids transfections following the manufacture’s protocols.

### Stable cell line construct

The short hairpin RNAs (shRNA) targeting human TIM sequences were as follows: sh-1, 5′-GCTAGAGATTGTCTCCCTT-3′, sh-2, 5′-CCAAATACATCCTGGGCAA-3′. The recombinant lentivirus containing oeACER2-TET-on plasmid, oeTIM plasmid or its empty plasmid was purchased from Obiotechnology Co., Ltd (Shanghai, China). The transfection steps were performed according to the manufacture’s protocols. Cells were plated at 60–70% confluence in 60 mm dishes. Then 1 × 10^6^ units recombinant lentivirus were added in the presence of 8 μg/ml Polybrene (H9268; Sigma-Aldrich, St. Louis, MO). Then cells were selected and cultured in the presence of 2 μg/ml puromycin.

### RNA isolation and quantitative real-time PCR (qRT-PCR)

Total RNA was extracted using TRIzol (Invitrogen) and RNA was reserve transcripted through PrimeScript RT-PCR kit (Takara) according to manufacturer’s instructions. RT-PCR was performed using SYBR Green Master Mix (Bimake) on the 7500 Real-time PCR system (Applied Biosystems) at the recommended thermal cycling settings: one initial cycle at 95 °C for 5 min followed by 40 cycles of 15 s at 95 °C and 34 s at 60 °C. β-actin was used as an internal control. All primers used in this study were listed in Supplementary Table 1.

### Western blotting

Western blotting was performed according to the previous study^18^. The antibodies used were shown as follows: TIM (ab72458; Abcom, Cambridge, USA), ACER2 (ARG66642, Arigo, Wuhan, China), ACER3 (PA5-39017, Thermo Fisher, MA, USA), Sp1 (ab227383, Abcom, Cambridge, USA), β-actin (M1210-2; Hua’an Biology, Chuzhou, China), Ki67 (GB13030; Servicebio, Wuhan, China).

### Cell counting kit-8 (CCK8) assay, colony formation assay

CCK-8 and colony formation assay were performed as previously described^19^.

### Subcutaneous and orthotopic xenograft model

In this study, athymic female nu/nu mice aged 6 weeks were used. Mice were manipulated and housed according to the criteria outlined in the Guide for the Care and Use of Laboratory Animals. All animal experiments were approved by the Institutional Animal Care and Use Committee of East China Normal University. Mice were assigned randomly to five groups. For subcutaneous xenograft model, a total number of 2 × 10^6^ target MCF7 cells in 100 μl PBS were injected into the right-back flank of mice. The tumor diameters were monitored every 5 days. After 1 week, all the groups were administered doxycycline (2 mg/mL) to activate the TET-on system. After 25 days, mice were sacrificed and tumor was isolated and weighted out. For orthotopic xenograft model, 1 × 10^6^ either shTIM or shNC MCF7 cells were injected with Matrigel into the left forth mammary fat pads of the mice. All mice were supplemented with estradiol pellets (0.72 mg, Innovative Research, Sarasota, FL, USA). The tumor diameters were monitored every 5 days. After 30 days, mice were sacrificed and tumor was isolated and weighted out. Tumor volume was calculated according to the formula: volume = length × width^2^/2.

### RNA sequencing

Total RNA was extracted using Trizol reagent following the manufacturer’s protocol. RNA integrity was evaluated using the Agilent 2100 Bioanalyzer (Agilent Technologies, Santa Clara, CA, USA). The samples with RNA Integrity Number (RIN) ≥ 7 were subjected to the subsequent analysis. The libraries were constructed using TruSeq Stranded mRNA LT Sample Prep Kit (Illumina, San Diego, CA, USA) according to the manufacturer’s instructions. Then these libraries were sequenced on the Illumina sequencing platform (Illumina HiSeq X Ten) and 150 bp paired-end reads were generated.

### KEGG analysis

KEGG analysis was performed using the KEGG pathway database (https://www.genome.jp/kegg/pathway.html). The different expression genes between siNC group and siTIM group were analyzed by KEGG. Furthermore, the *p* value was calculated.

### Intracellular S1P measurement

Cells (1 × 10^6^ cells) were cultured 72 h in 60 mm dishes after indicated treatments. Then the medium was removed and cells were washed twice with ice-cold PBS. Cells were scraped with ice-cold PBS and cell pellets were separated by centrifugation at 1000 rpm for 5 min. Cell pellets were stored at −80 °C for further S1P extraction. S1P extraction and quantitation of S1P were performed by HPLC-MS using multiple reaction monitoring (MRM) as previously described^20^.

### Immunohistochemistry (IHC) staining

We examined the expression of TIM with immunohistochemistry (IHC) using a human breast cancer tissue microarray containing 93 breast cancer samples from Service Biotechology Co., Ltd. (Wuhan, China). IHC staining was performed according to the protocol described previously^18^. Anti-TIM (1:200, ab72458; Abcom, Cambridge, USA) antibodies were used. The percentage of positive cell scores was divided into 0 (0–5%), 1 (6–35%), 2 (36–70%), and 3 (more than 70%); The intensity of protein expression was determined as 0 (no staining),1 (weakly staining), 2 (moderately staining) and 3 (strongly staining). The final score was calculated using the percentage score × staining intensity score as follows: 0(−), 2–3 (+), 4–6 (++), and >6 (+++). The final score <4 was defined as low expression and final score ≥4 was defined as high expression. These scores were determined by two independent pathologists in a blinded manner.

### Immunocytochemistry (ICC)

Cells were plated in 12-well chambers (Ibidi, Martinsried, Germany). We fixed cells with 4% polyformaldehyde (PFA) for 15 min (at room temperature) and permeabilized with 1% TrixonX-100 for 2 min. Cells were blocked with 1% bovine serum albumin (BSA) for 1 h (at room temperature). Then cells were incubated with TIM antibody (1:200), Sp1 antibody (1:200) overnight (at 4 °C), and followed by species-matched secondary antibodies (1:200) for 1 h (at room temperature). We stained the nucleus with DAPI (D9542; Sigma-Aldrich, St. Louis, MO) for 2 min (at room temperature). Then, we acquired the images with confocal microscopy (LSM510, Carl Zeiss, Oberkochen, Germany).

### Oxidative phosphorylation analysis

Oxygen consumption rate (OCR) of MCF7 and T47D target breast cancer cells was measured using Seahorse XF96 extracellular flux analyzer (Seahorse Bioscience, Agilent) according to the manufacturer’s instructions. In this study, a total number of 3 × 10^4^ cells were seeded in a XF96-well plate with indicated treatments. The media was removed one hour before the assay and the cells were washed three times with PBS. Then the cells were replaced with base medium (non-buffered DMEM supplemented with 25 mM glucose). For OCR, 1.5 μM oligomycin, 2 μM FCCP, 0.5 μM rotenone, and 0.5 μM actinycin A were injected sequentially into the wells. The assay was performed with four technical replicates. Spare respiratory capacity = maximum respiration − basal respiration.

### Co-immunoprecipitation (Co-IP) assay

Total cellular protein was extracted by RIPA lysis buffer (Beyotime, Shanghai, China). Cell lysates were subjected to immunoprecipitation with either anti-Sp1 (anti-TIM) antibody or control IgG. Then the immunoblotting was performed with anti-TIM (anti-Sp1) antibody.

### Chromatin immunoprecipitation (ChIP) assay

ChIP assays were performed using the ChIP assay kit (Pierce Agarose ChIP Kit, Thermo Fisher). We quantified the DNA–protein complexes which was immunoprecipitated with anti-Sp1 (9389, Cell Signaling Technologies) or control IgG (Cell Signaling) from the sonicated cell lysates with Premix Taq (Cell Signaling). The primer used in the process of ChIP were listed in Supplementary Table 2.

### Luciferase reporter assay

Luciferase reporter assay was performed as described in our previous study^19^. In brief, wild-type or Sp1-overexpressed MCF7 cells were co-transfected with pGL3B-Promotor vectors and pRL-TK Renilla plasmids. The Dual-Luciferase Reporter Assay System (Promega) was performed to analyze the luciferase activity. The wild and mutant ACER2 promoter regions were listed in Supplementary Table 3.

### Database analysis

Kaplan–Meier analysis of breast cancer patients was referenced from the KM plot database (http://kmplot.com/analysis). GEO databases (GSE3744, GSE109169) were used to compare TIM expression levels between breast cancer tissue and normal tissue.

### Statistical analysis

There are no data excluded from analysis. In regard to experimental grouping, the data analysts were blind. According to previous research, we confirm the sample size and power analysis of every experiment^21^. Multivariate logistic regression was used for the association between clinicopathological factors with pathologic complete response (pCR). Category variables were performed by the Pearson chi-square test. The Student’s *t*-test was used for continuous variables. In addition, for every figure, the statistical tests are appropriate. All error bars in this study was represented the mean ± SD from triplicate experiments performed in a parallel manner. Values of *p* < 0.05 were considered statistically significant. All statistics were carried out using GraphPad Prism 10.0, STATA Statistics SE 14.

## Results

### High expression of TIM predicts poor prognosis in ER-positive breast cancer

**Fig. 1:**
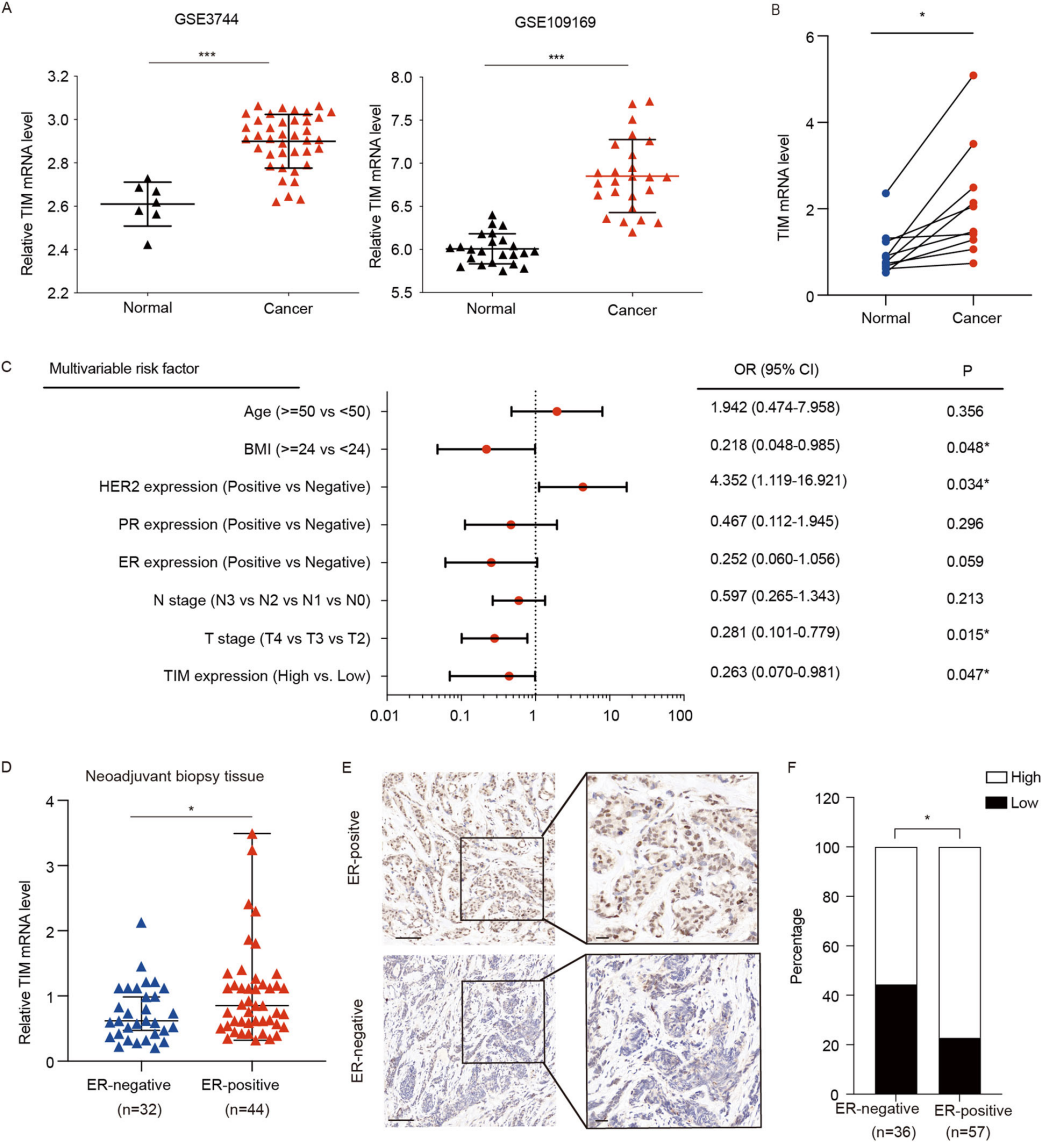
TIM expression is increased in breast cancer especially in ER-positive cancer and is correlated with poor prognosis. **a** TIM expression analysis in tumor tissue compared to paired non-tumor tissue in breast cancer using two independent GEO cohort (GSE3744, GSE10969). Values are means ± SD, ***p < 0.001 (Student’s *t*-test). **b** Relative mRNA level of tumor and paired normal tissue in patients with breast cancer from Renji cohort. Values are means ± SD, *p < 0.05 (Student’s *t*-test). **c** Multivariate logistic regression analysis of clinicopathological factors for pathologic complete response (pCR) applied the neoadjuvant biopsy tissue from 76 patients with breast cancer. OR: odds ratio; CI: confidence interval. BMI: body mass index. **d** Relative TIM mRNA level of neoadjuvant biopsy tissue including 44 ER-positive and 32 ER-negative patients. **e** Representative IHC staining for TIM in ER-positive (*n* = 36) and ER-negative (*n* = 57) patients from human breast cancer microarray. Scale bar is 50 mm. **f** The percentage of tissue cores displaying low or high TIM staining in ER-positive (*n* = 36) and ER-negative (*n* = 57) lesions from human breast cancer microarray. *p < 0.05 (Fisher’ s exact). **g** Kaplan–Meier analysis of the association between TIM and overall survival (OS), relapse-free survival (RFS), and distance metastasis-free survival (DMFS) of ER-positive breast cancer patients from KM plot database.

The gene expression data from GEO databases (GSE3744, GSE109169) showed that TIM mRNA expression was significantly upregulated in breast cancer tissues compared to the normal tissues (Fig. 1a). To further verify the TIM expression in breast cancer, we examined mRNA levels of *TIM* in 10 paired cancer and corresponding adjacent tissues, revealing enhanced TIM expression in cancer tissue (Fig. 1b). In addition, multivariable logistic regression analyses showed that body mass index (BMI), HER2 expression level, T stage, and TIM expression level were significantly associated with pCR by using 76 neoadjuvant biopsy tissues, including 44 ER-positive and 32 ER-negative tissues from Renji Hospital (Fig. 1c). We further compared *TIM* level in ER-positive and ER-negative tissues showed that the expression of *TIM* in ER-positive breast cancer was significantly higher than in ER-negative subtype (Fig. 1d). These observations were further confirmed in the human breast cancer microarray (Fig. 1e, f). Furthermore, the Kaplan–Meier analysis revealed that high expression of TIM in cancer tissues was associated with poor prognosis, especially in the ER-positive breast cancer patients (Fig. 1g, S1a). However, there is no relationship between TIM expression and prognosis in the ER-negative breast cancer patients (Fig. S1b). Taken together, these data revealed that TIM was highly expressed and its high expression predicted poor prognosis in breast cancer, especially in ER-positive breast cancer.

### TIM enhances ER-positive breast cancer cell growth both in vitro and in vivo

**Fig. 2:**
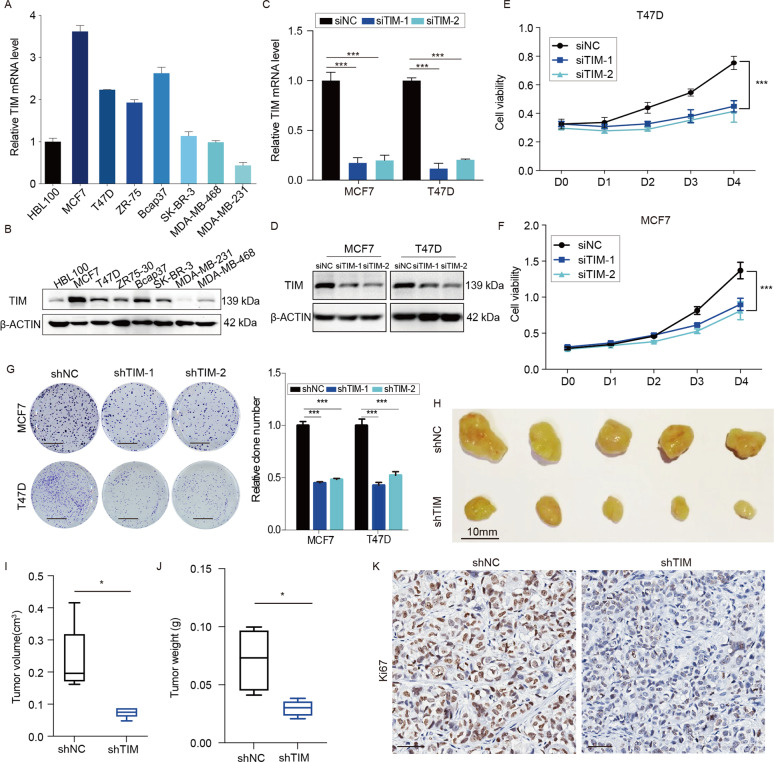
TIM knockdown inhibits breast cancer cell proliferation. **a**, **b** Relative mRNA and protein expression level of TIM in seven breast cancer cell lines and normal mammary epithelial cell line HBL100. Values are means ± SD. **c**, **d** The interference efficiency of TIM via small interfering (siRNA) in MCF7 and T47D cells. Values are means ± SD, ***p < 0.001 (Student’s *t*-test). **e**, **f** Cell viability of MCF7 and T47D cells interfered by siRNA. Values are means ± SD, ***p < 0.001 (Student’s *t*-test). **g** Colony formation assay of MCF7 and T47D stably interfered by shRNA. Values are means ± SD, ***p < 0.001 (Student’s *t*-test). Scale bar is 5 mm. **h** Orthotopic xenograft transplanted with shNC and shTIM/MCF7 cells (*n* = 5). Scale bar is 10 mm. **i** Tumor volume at day 30 was measured in each group. Values are means ± SD, **p < 0.01 (Student’s *t*-test). **j** Tumor weight at day 30 was measured in each group. Values are means ± SD, *p < 0.05. **k** Representative images of Ki67 staining in orthotopic xenograft tissue from shNC and shTIM mice. Scale bar is 50 mm.

To clarify the biological functions of TIM in breast cancer, the TIM expression in ER-positive breast cancer cell lines MCF7, T47D, and ZR75-30, ER-negative breast cancer cell lines Bcap37, SH-BR-3, MDA-MB-231, and MDA-MB-468, and normal mammary epithelial cell line HBL100 was tested by both qPCR and western blotting (WB) (Fig. 2a, b). The results showed high TIM expression in most ER-positive breast cancer cell lines and therefore the ER-positive cell lines MCF7 and T47D were chosen for subsequent experiments. The interfering efficient of TIM via siRNA and shRNA were detected by both qPCR and WB in MCF7 and T47D cells, respectively (Fig. 2c, d, S2a,b). Knockdown of TIM inhibited cell proliferation and colony formation (Fig. 2e–g). To explore the effect of TIM on the proliferation of breast cancer cells in vivo, the orthotopic xenograft mouse model was performed. The knockdown of TIM significantly inhibited the tumor growth (Fig. 2h–j, S2c). IHC staining results revealed that Ki67 staining was decreased in the orthotopic xenograft tumors of TIM-knockdown group compared to the control group (Fig. 2k). These data suggested that TIM promoted tumor growth in ER-positive breast cancer both in vitro and in vivo.

### TIM promotes the S1P biosynthesis in breast cancer cells via regulating ACER2

**Fig. 3:**
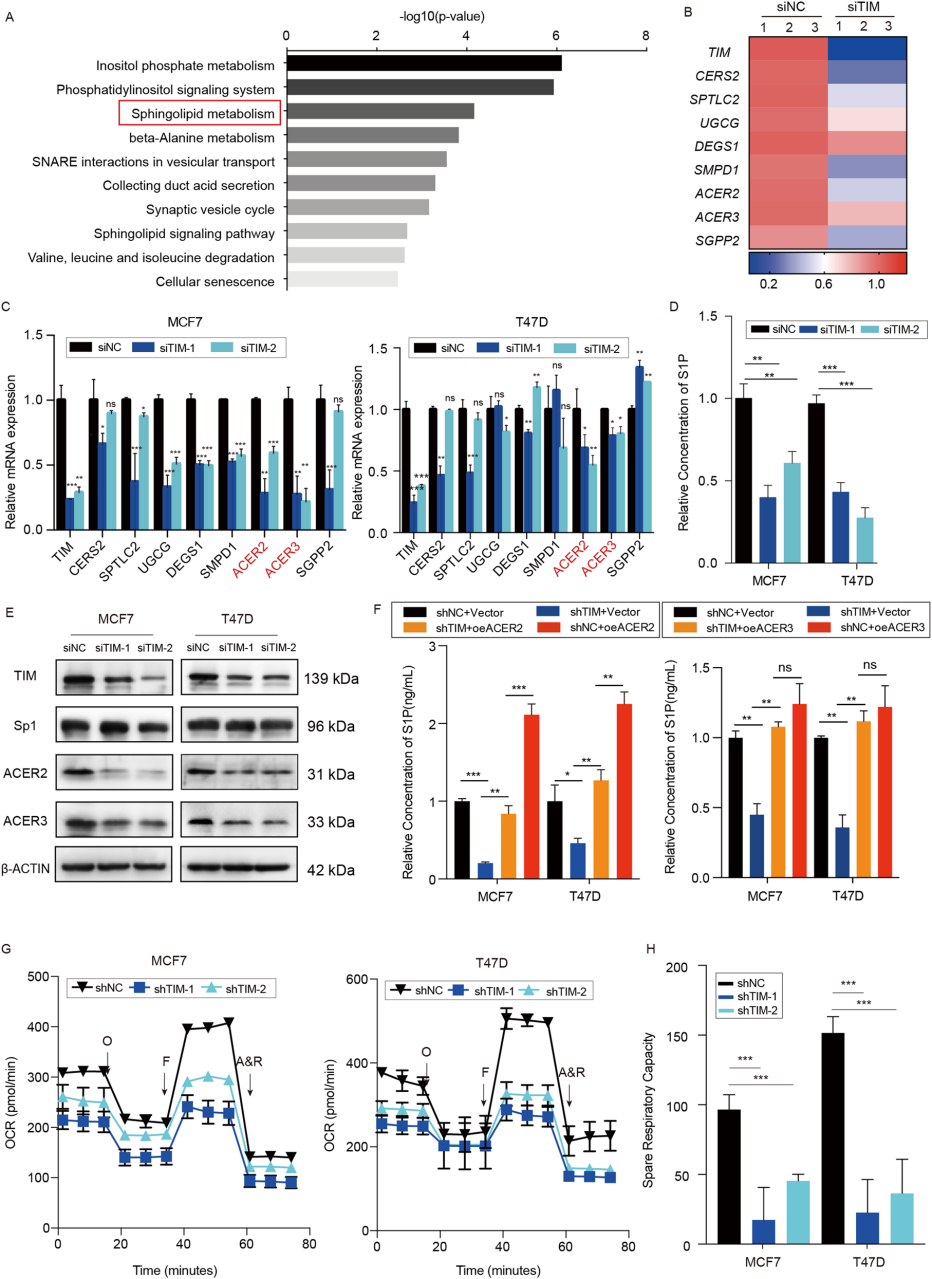
TIM regulates S1P synthesis via modulating ACER2 expression. **a** KEGG pathway analysis using hallmark gene sets was performed to compare the MCF7-siNC group and siTIM group. **b** Heatmap showing the expression of key enzymes in sphingolipid metabolism across MCF7 siNC samples and siTIM samples. **c** Relative mRNA levels of sphingolipid metabolism-related genes in siNC or siTIM MCF7 (left) and T47D (right) cells. Values are means ± SD, *p < 0.05; **p < 0.01; ***p < 0.001 (Student’s *t-*test). **d** Relative S1P concentration in siTIM or siNCMCF7 and T47D cells (*n* = 3). Values are means ± SD, **p < 0.01; ***p < 0.001 (Student’s *t*-test). **e** ACER2, ACER3, and Sp1 protein expression level of MCF7 and T47D cells in siTIM sample and siNC sample. **f** Relative S1P concentration of MCF7 and T47D cells in different groups (shNC+vector, shNC+oeACER2, shTIM+oeACER2, shTIM+vector) were measured. Values are means ± SD, ns: no significance; *p < 0.05; **p < 0.01; ***p < 0.001 (Student’s *t*-test). **g** The OCR of shTIM or shNC MCF7 and T47D cells were determined (*n* = 3). O: Oligomycin, F: FCCP; A&R: antimycin A/rotenone. **h** Spare respiratory capacity of shTIM or shNC MCF7 and T47D cells were analyzed. Values are means ± SD, ***p < 0.001 (Student’s *t*-test).

In order to study the underlying mechanism by which TIM promoted breast cancer cell growth, we performed transcriptome sequencing (RNA-seq) to find out the difference between TIM-knockdown and control MCF7 cells. KEGG analysis indicated that differentially expressed genes were mostly enriched at sphingolipid metabolism pathway (Fig. 3a). The key genes in the sphingolipid metabolism pathway were significantly downregulated in TIM-knockdown cells compared to control cells (Fig. 3b). These results were further verified by qPCR and we found that the expression of ACER2 and ACER3 were most obviously downregulated in both MCF7 and T47D cells (Fig. 3c, e). Previous researches have shown that ACER2 and ACER3 were the key genes in the biosynthesis of S1P^22^. To gain further insight into the effect of TIM on the biosynthesis of S1P in breast cancer cells, HPLC-MS was performed in both TIM-knockdown and control MCF7 and T47D cells to compare the level of S1P, which is one of the most vital final products of the sphingolipid metabolism. The result demonstrated that the level of S1P was significantly decreased in TIM-knockdown cells compared to control ones (Fig. 3d, S3a). To explore whether TIM regulates the biosynthesis of S1P via regulating the expression level of ACER2 and ACER3, HPLC-MS showed that ACER2 not ACER3 overexpression abolished the decrease of S1P via TIM knockdown (Fig. 3f). Collectively, these findings demonstrated that TIM facilitated S1P biosynthesis in breast cancer cells by modulating ACER2 expression. Previous study has shown that S1P regulates mitochondrial oxidative phosphorylation in a receptor-independent manner^23^. To investigate whether the mitochondrial oxidative phosphorylation was affected by TIM expression, the OCR was measured. The results showed that TIM knockdown significantly reduced the oxidative phosphorylation compared to the control cells (Fig. 3g, h). Taken together, the data suggested that TIM regulated S1P synthesis via modulating ACER2 expression.

### ACER2 is responsible for TIM-mediated cell growth in breast cancer cells

**Fig. 4:**
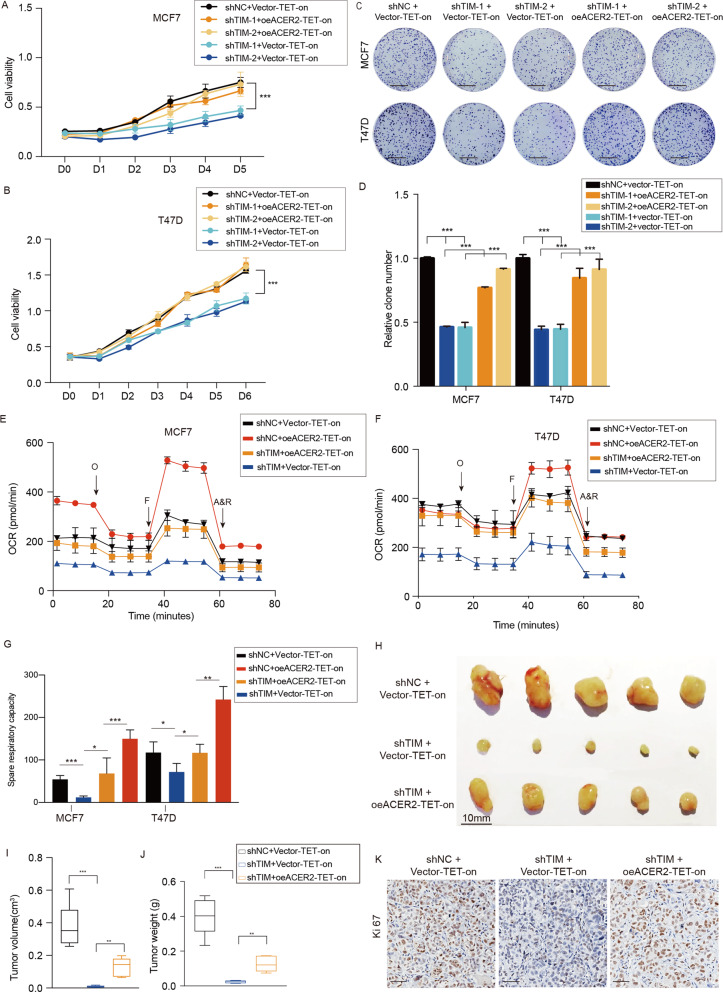
ACER2 overexpression partly reverses the effect of TIM knockdown in breast cancer cells. **a**, **b** Cell viability of MCF7 and T47D cells under the indicated treatments. Values are means ± SD, ***p < 0.001 (Student’s *t*-test). **c**, **d** Colony formation assay of MCF7 and T47D cells under the indicated treatments. Values are means ± SD, ***p < 0.001 (Student’s *t*-test). Scale bar is 5 mm. **e**, **f** The OCR of MCF7 and T47D cells under the indicated treatments (*n* = 3). O: Oligomycin, F: FCCP; A&R: antimycin A/rotenone. **g** Spare respiratory capacity of MCF7 and T47D cells under the indicated treatments. Values are means ± SD, *p < 0.05; **p < 0.01; ***p < 0.001 (Student’s *t*-test). **h** Morphologic characteristic of subcutaneous xenograft tumors from MCF7/shNC + vector-TET-on group, MCF7/shTIM+vector-TET-on group, MCF7/shTIM + oeACER2-TET-on group (*n* = 5). Scale bar is 10 mm. **i** Tumor volume at day 25 was measured in each group. Values are means ± SD, **p < 0.01 (Student’s *t*-test). **j** Tumor weight at day 25 was measured in each group. Values are means ± SD, *p < 0.05. **k** Representative images of Ki67 staining in xenograft from each group. Scale bar is 50 mm.

We next investigated whether overexpression of ACER2 could abolish the inhibition of TIM knockdown on the proliferation and the mitochondrial respiration of breast cancer cells. We utilized the ACER2-TET-on in control and TIM knockdown cells. As expected, overexpressing ACER2 in TIM-knockdown breast cancer cell rescued the cell growth and the ability of colony formation (Fig. 4a–d). In addition, we silenced ACER2 in the control and TIM knockdown cells and performed the CCK8 and colony formation assay. The results showed that the cell growth and the ability of colony formation were further suppressed when TIM and ACER2 were knockdown at the same time (S4. b-d). Consistently, ACER2 overexpression also reversed the mitochondrial respiration in the TIM knockdown cells (Fig. 4e–g). To verify these effects in vivo, the subcutaneous xenograft mouse model was performed. The results showed that ACER2 overexpression partly restored the anti-growth effect caused by TIM knockdown (Fig. 4h–k). Taken together, these data indicated that the promotive effect of TIM on proliferation and mitochondrial respiration were primarily mediated by ACER2 in breast cancer cells.

### TIM, as a coactivator of Sp1, transcriptionally regulates ACER2 in breast cancer cells

**Fig. 5:**
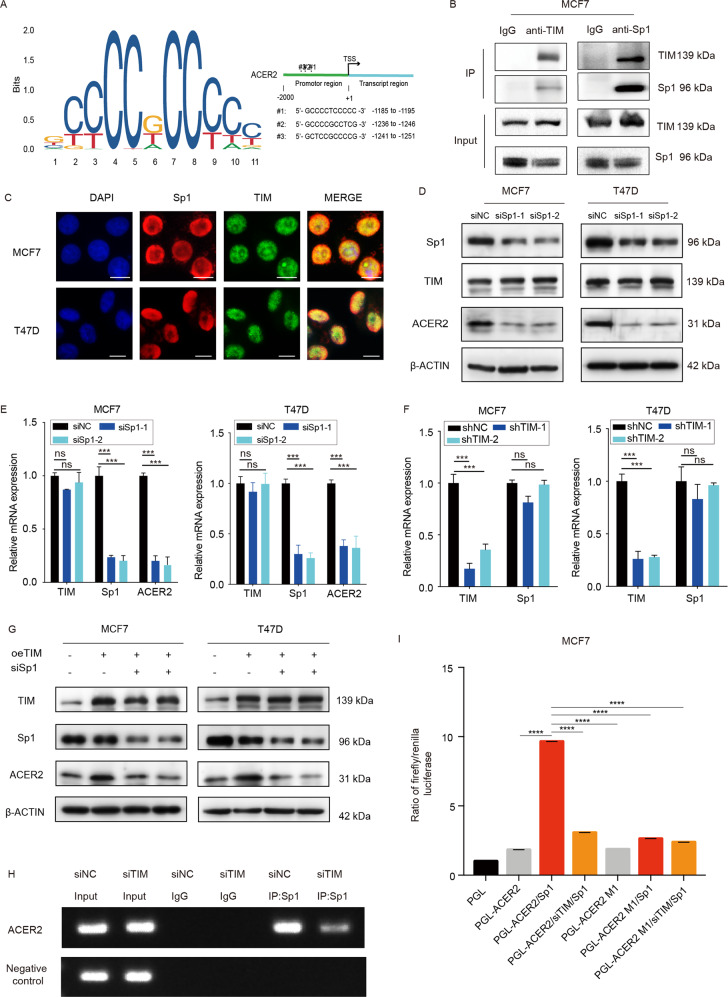
TIM, as coactivator of Sp1, transcriptionally regulates ACER2 in breast cancer cells. **a** Jasper database predicts the potential Sp1 binding sequences in ACER2 promotor region. **b** Co-immunoprecipitation of TIM and Sp1 in MCF7 cells. **c** Immunocytochemistry staining of TIM and Sp1 in MCF7 and T47D cells. TIM is shown in red, Sp1 is shown in green and cell nuclei were stained with DAPI in blue. Scale bar is 10 mm. **d** TIM and ACER2 expression level of MCF7 and T47D cells in Sp1 knockdown and control groups. **e** Relative mRNA levels of TIM, Sp1 and ACER2 in MCF7 and T47D cells knockdown by siSp1. Values are means ± SD, ns: no significance; ***p < 0.001 (Student’s *t*-test). **f** Relative mRNA levels of TIM and Sp1 in MCF7 and T47D cells knockdown by siTIM. Values are means ± SD, ns: no significance; ***p < 0.001 (Student’s *t*-test). **g** ACER2 protein expression of MCF7 and T47D cells in different groups (vector + siNC, oeTIM + siNC, oeTIM + siSp1-1, oeTIM + siSp1-2). **h** ChIP-PCR analysis of Sp1 binding to ACER2 promotor region in MCF7/siTIM cells and control cells. **i** Luciferase activities of Sp1 overexpression, TIM knockdown+Sp1 overexpression, and their control cells in luciferase reporter plasmid containing wild-type and mutant 1 (M1) ACER2 promoter. The data shown are mean ± SD, ****p < 0.0001 (Student’s *t*-test).

**Fig. 6:**
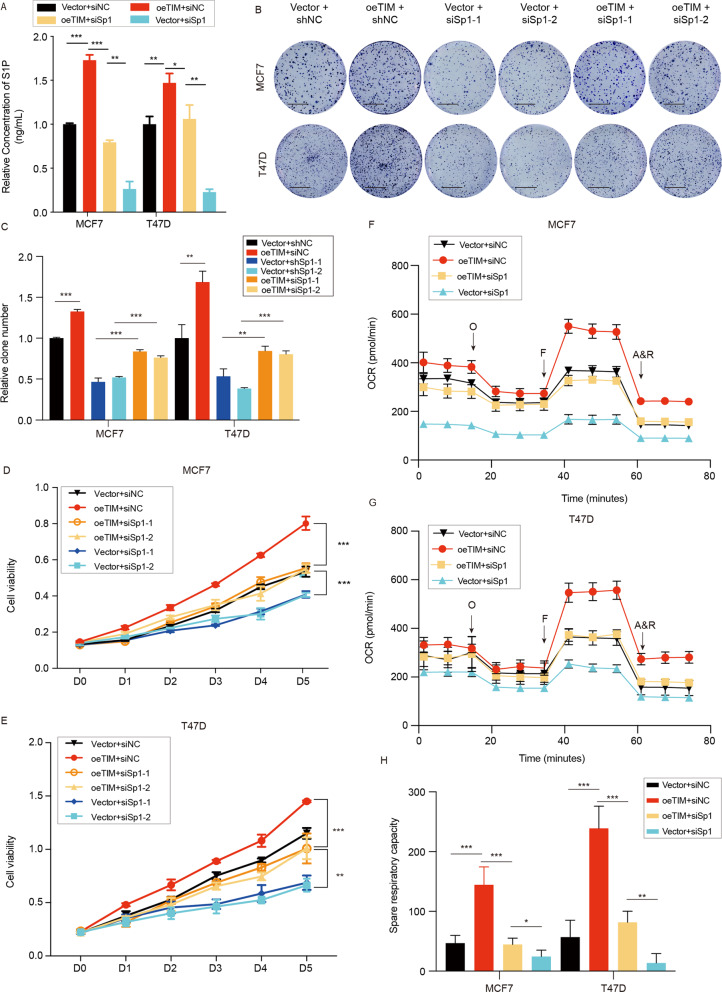
Sp1 knockdown partly rescues the effect of TIM overexpression in breast cancer cells. **a** Relative S1P concentration of MCF7 and T47D cells in different groups (vector+siNC, oeTIM+siNC, oeTIM+siSp1, vector+siSp1) were measured. Values are means ± SD, *p < 0.05; **p < 0.01; ***p < 0.001 (Student’s *t*-test). **b**, **c** Colony formation assay of MCF7 and T47D cells under the indicated treatments. Values are means ± SD, **p < 0.01, ***p < 0.001 (Student’s *t*-test). Scale bar is 5 mm. **d**, **e** Cell viability of MCF7 and T47D cells under the indicated treatments. Values are means ± SD, **p < 0.01, ***p < 0.001 (Student’s *t*-test). **f**, **g** The OCR of MCF7 and T47D cells under the indicated treatment (*n* = 3). O: Oligomycin, F: FCCP; A&R: antimycin A/rotenone. **h** Spare respiratory capacity of MCF7 and T47D cells under indicated treatment. Values are means ± SD, *p < 0.05; **p < 0.01; ***p < 0.001 (Student’s *t*-test).

Previous researches indicate that TIM, as a coactivator, interacts with Per2 to regulate the expression of Bmal1 and Clock^12^. However, Per2 has no binding site in ACER2 promotor according to Jasper database. We speculated that TIM might act as a coactivator of other transcription factor to regulate the expression of ACER2. To find out which transcription factor could interact with TIM, we searched for the potential candidates which can bind to TIM in Biogrid database. Among 81 potential candidates, we found out that Sp1 was the only transcription factor which could bind to the promotor of ACER2 through Jasper database (Fig. 5a). We further confirmed the physical interaction between TIM and Sp1 by Co-IP (Fig. 5b). Moreover, we observed the colocalization of TIM with Sp1 in the breast cancer cells via ICC (Fig. 5c). We then confirmed that Sp1 could regulate the expression of ACER2 but no TIM in both mRNA and protein level (Fig. 5d, e). In addition, we found that TIM expression did not influence the Sp1 expression level in MCF7 and T47D cells. (Figs. 5f, 3e). To confirm whether Sp1 together with TIM contributes to the regulation of ACER2, we examined the alteration of ACER2 expression with the knockdown of Sp1 in the TIM-overexpressed cells (Fig. 5g, S4a). The result indicated that knockdown of Sp1 could inhibit the upregulation of ACER2 stimulated by TIM overexpression. The ChIP-PCR assay confirmed that ACER2 was the target gene of TIM-Sp1 (Fig. 5h, S5. a, b). In addition, luciferase reporter assay was performed to directly demonstrate that the wild-type ACER2 promotor but not mutant construct was activated by Sp1 overexpression and knockdown TIM impaired the transcriptional activity of Sp1. (Fig. 5i). Then we found that Sp1 knockdown impaired the TIM-induced increase of S1P (Fig. 6a). Knockdown of Sp1 partially abolished the promotion of both cell proliferation and mitochondrial respiration caused by the overexpression of TIM (Fig. 6b–h). Taken together, these data suggested that TIM, which interacted with Sp1, upregulated the expression of ACER2 and further modulated the synthesis of S1P.

## Discussion

Recent studies reveal that circadian clock genes are important in tumorigenesis, metastasis, and chemotherapeutic resistance^24–26^. In this study, we identify a novel function of a circadian clock gene TIM in the ER-positive breast cancer progression. High expression of TIM in breast cancer predicts poor prognosis and TIM regulates tumor cell growth and sphingolipid metabolism through Sp1/ACER2/S1P axis.

In our study, transcriptome sequencing analysis demonstrates that TIM regulates sphingolipid metabolism, indicating that the growth-promoting roles of TIM may largely dependent on sphingolipid metabolism. As the structural molecules of cell membrane, sphingolipids are essential for maintaining barrier function and fluidity^27^. Furthermore, sphingolipids play important roles on regulating various physiological and pathological processes such as tumor growth, invasion, and metastasis through regulating molecular signaling functions^28,29^. S1P is one of the most central bioactive products in sphingosine metabolism which regulates diverse biological functions mainly by binding to its five specific G protein-coupled receptors^30–32^. We show for the first that TIM regulates the synthesis of S1P.

Next, we uncover that TIM regulates S1P biosynthesis by controlling the expression of ACER2. Previous research has demonstrated that ACER2 plays a vital role in the maintenance of S1P in plasma by controlling the production of sphingosine (SPH) in hematopoietic cells^22^. Several researches indicate that ACER2 is associated with proliferation, apoptosis, DNA damage response, and autophagy in diverse cancer cells^33–37^. In this study, we demonstrate that the decrease of tumor growth induced by TIM knockdown is dependent on the expression of ACER2. As a coactivator, TIM interacts with period circadian regulator 2 (Per2) to regulate the expression of Bmal1 and Clock^12^. However, there are no binding sites of Per2 in ACER2 promotor region. In order to find the transcription factor which mediates the regulation of ACER2 by TIM, we searched for the potential TIM interactive protein in the Biogrid database. Then we found that TIM may interact with Sp1 and Sp1 has potential binding site in ACER2 promotor region according to Jasper database. Sp1 is a well-known member of the transcription factor family. Sp1 activates the transcriptions of a large number of genes that contain CG-rich promoters and Sp1 target genes play pivotal roles in tumor growth, apoptosis, and tumorigenesis^38^. In addition, the activity of Sp1 is regulated by interacting with other protein factors and post-translational modifications^39^. However, the role of Sp1 in breast cancer is still controversial. It is reported that Sp1 overexpression predicts poor prognosis in some breast cancer patients while Jumonji domain-containing 2A-dependent inhibition Sp1 promotes metastasis in advanced breast cancer^40,41^. In our study, we find that TIM, as coactivator of Sp1, transcriptionally regulates ACER2. However, TIM and Sp1 do not regulate each other’s expression, neither mRNA nor protein level.

Our results also reveal that TIM regulates the mitochondrial respiration in breast cancer cells. Previous research has shown that S1P maintains naive T-cell mitochondrial content and function to provide energy for cells to survive^42–45^. In addition, S1P has also been found to regulate oxidative phosphorylation of mitochondria by interacting with prohibitin 2^23^. Consistent with previous studies, we also reveal that overexpressing either ACER2 or Sp1 restore mitochondrial respiration caused by TIM knockdown and we find that TIM knockdown inhibits the activity of mitochondrial complex IV (S3b). However, the potential molecular mechanisms between TIM and mitochondrial respiration need to be further explored. In addition, the direct evidence is still missing that S1P is responsible for the decrease of tumor growth and cell mitochondrial respiration which caused by TIM knockdown and needs to be further verified. Considering of sphingosine kinase 2 (SPHK2) is the downstream of ACER2 and the phosphorylation of sphingosine into S1P is catalyzed by SPHK2^23^. In further studies, SPHK2 inhibitor needs to be used to prove that S1P is responsible for the promotion of tumor growth and cell mitochondrial respiration caused by ACER2 overexpression.

In conclusion, our research provides a new enlightenment into the molecular mechanisms of circadian clock gene TIM in the development of ER-positive breast cancer. Our results demonstrate that TIM interacts with SP1 to regulate the expression of ACER2 and then modulates the synthesis of S1P. In addition, TIM enhances the mitochondrial respiration to promote cell proliferation by regulating the level of S1P. Therefore, targeting TIM might constitute a new approach for therapeutic intervention of breast cancer.

## Supplementary information

Supplemental figure 1

Supplemental figure 2

Supplemental figure 3

Supplemental figure 4

Supplemental figure 5

Supplemental figure 6

Supplemental table

Supplemental Figure legend
