# Supplementary figures and images for: TIMELESS regulates sphingolipid metabolism and tumor cell growth through Sp1/ACER2/S1P axis in ER-positive breast cancer
Source: Cell Death Dis. 2020 Oct 22;11(10):892. doi: 10.1038/s41419-020-03106-4 (PMC7581802; doi:10.1038/s41419-020-03106-4)

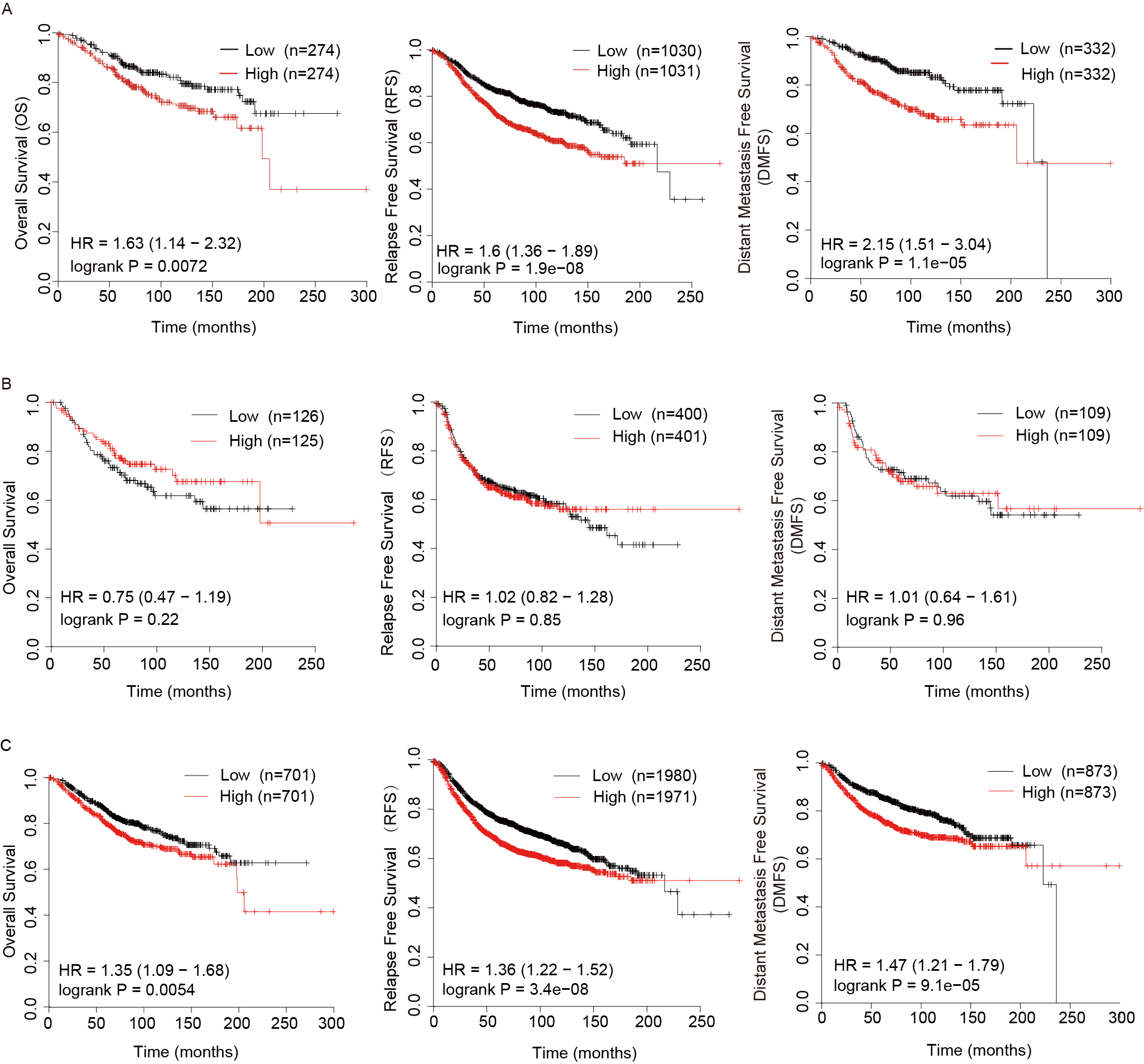

Supplement: Supplementary file 1 — Supplemental figure 1 [file 41419_2020_3106_MOESM1_ESM.png]

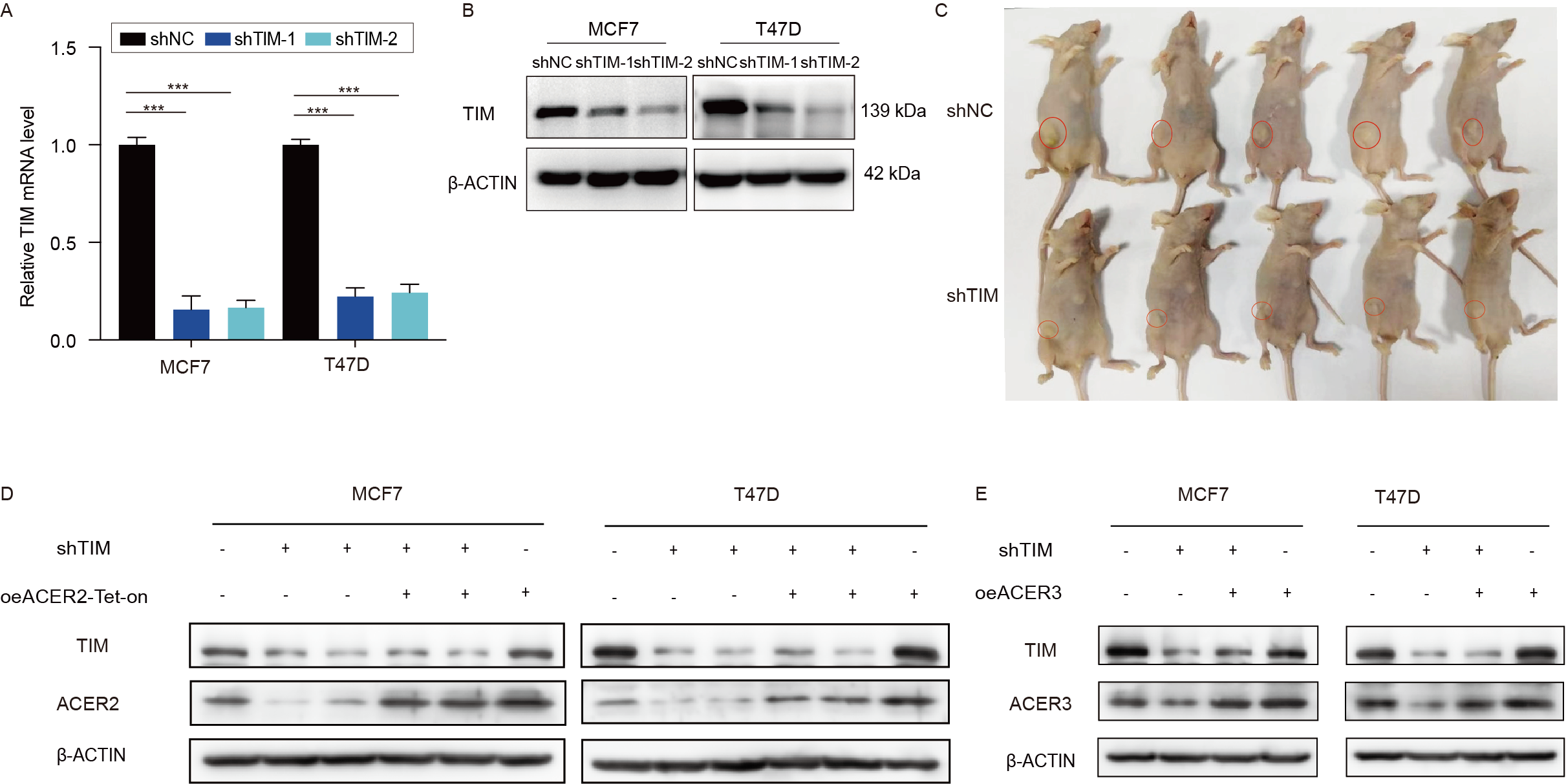

Supplement: Supplementary file 2 — Supplemental figure 2 [file 41419_2020_3106_MOESM2_ESM.png]

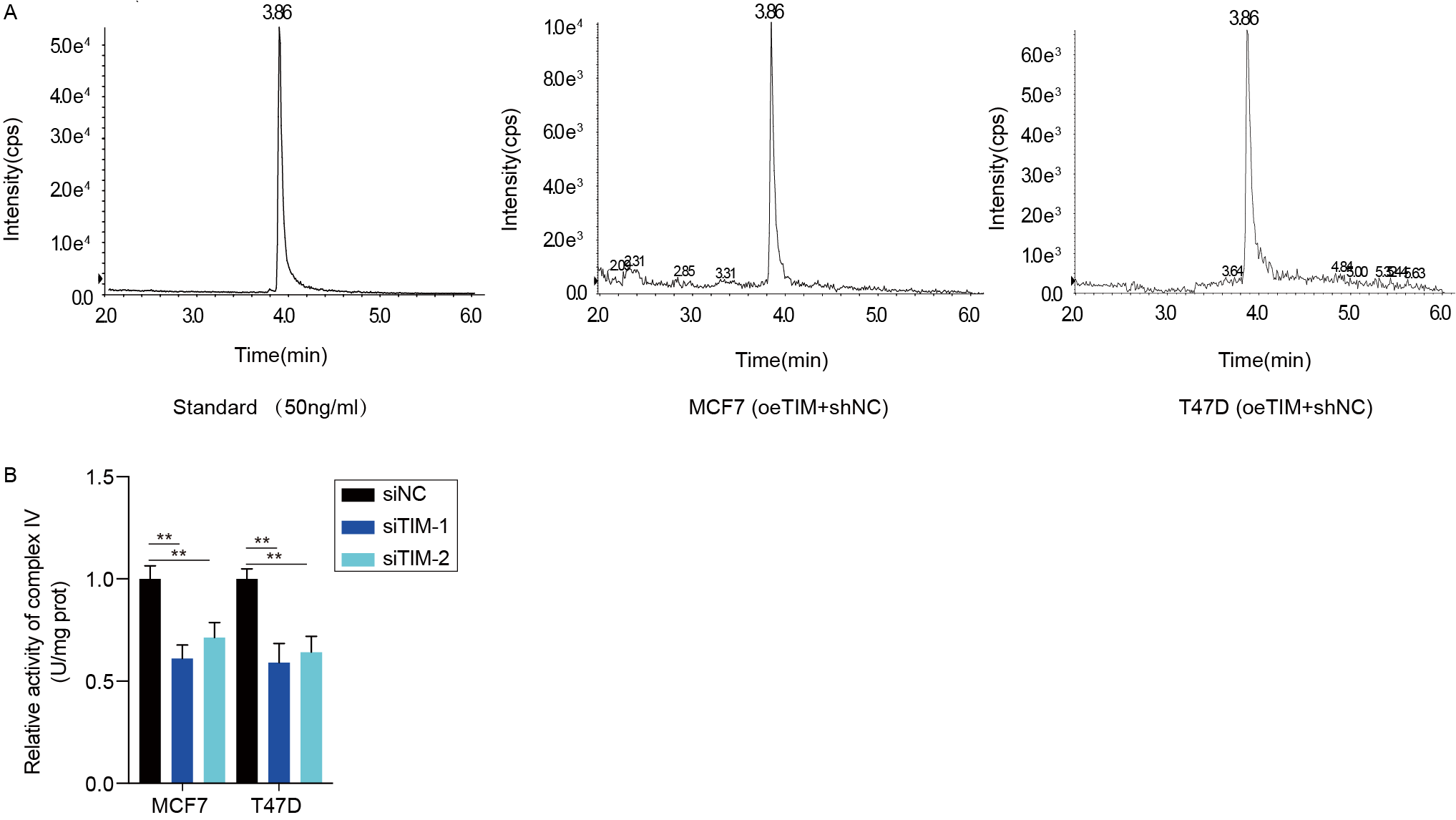

Supplement: Supplementary file 3 — Supplemental figure 3 [file 41419_2020_3106_MOESM3_ESM.png]

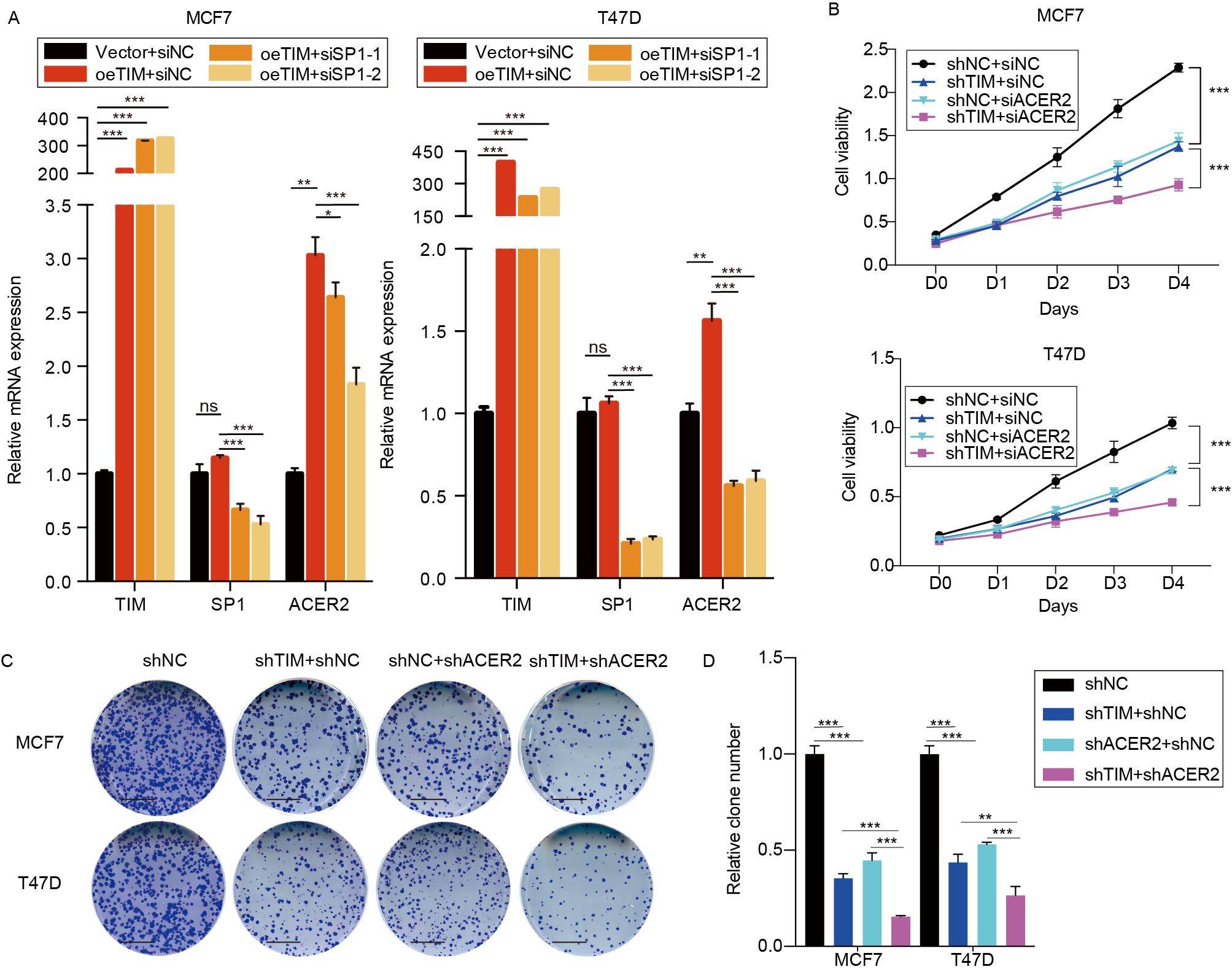

Supplement: Supplementary file 4 — Supplemental figure 4 [file 41419_2020_3106_MOESM4_ESM.png]

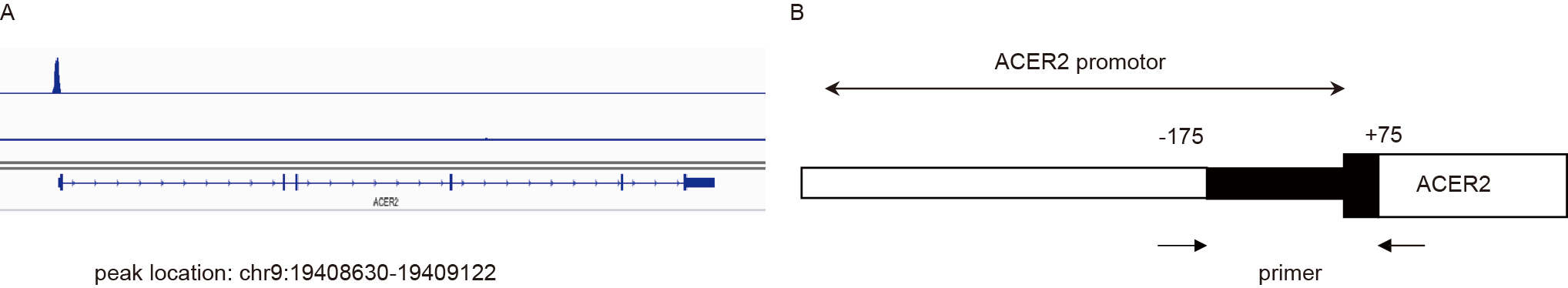

Supplement: Supplementary file 5 — Supplemental figure 5 [file 41419_2020_3106_MOESM5_ESM.png]

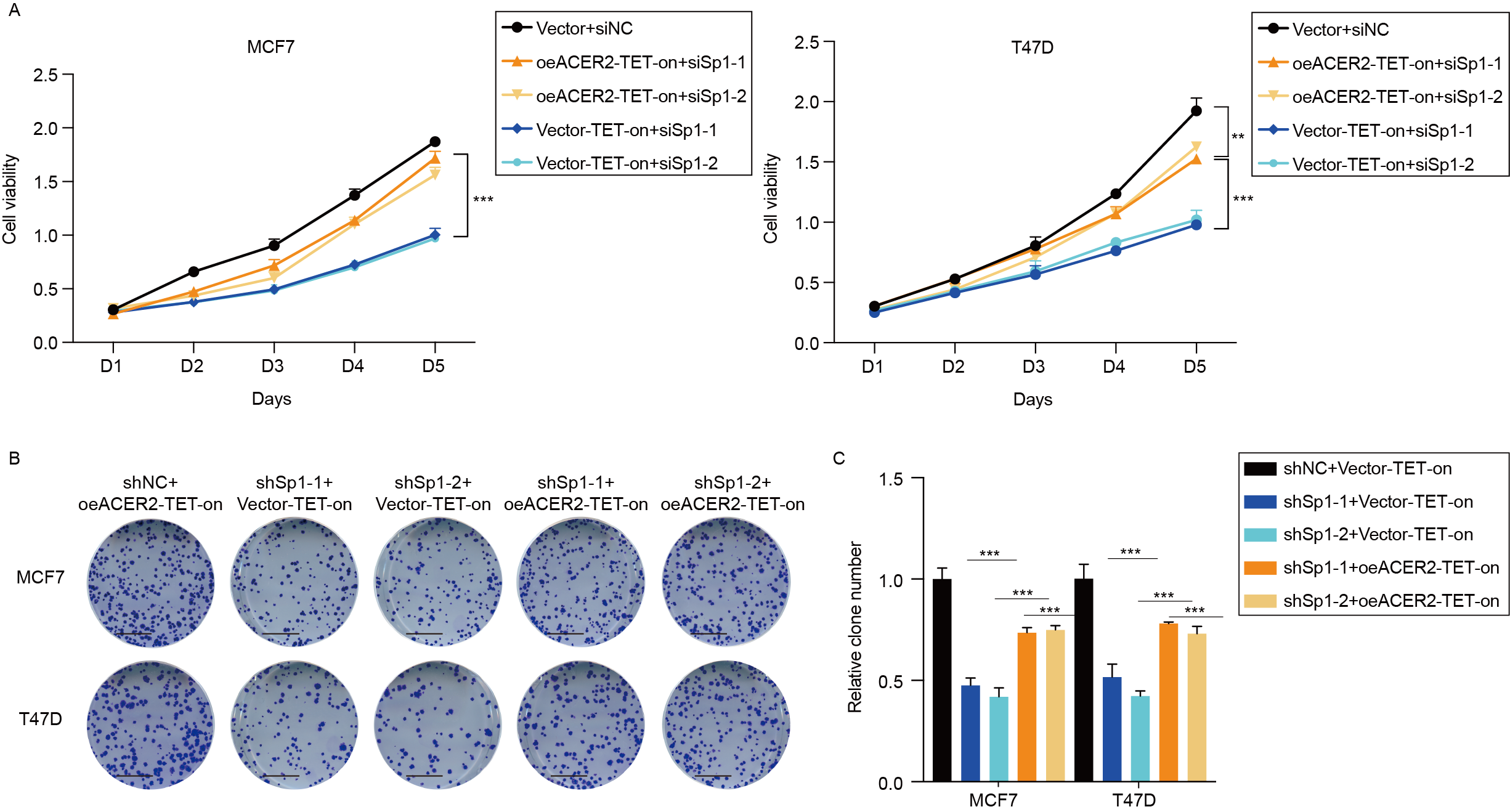

Supplement: Supplementary file 6 — Supplemental figure 6 [file 41419_2020_3106_MOESM6_ESM.png]
