# Supplementary material for: TIMELESS regulates sphingolipid metabolism and tumor cell growth through Sp1/ACER2/S1P axis in ER-positive breast cancer
Source: Cell Death Dis. 2020 Oct 22;11(10):892. doi: 10.1038/s41419-020-03106-4 (PMC7581802; doi:10.1038/s41419-020-03106-4)
Supplement: Supplementary file 7 — Supplemental table [file 41419_2020_3106_MOESM7_ESM.docx]

**Supplementary Table 1 Primers used for quantitative PCR in this study**

| Primer Name | Sequence (5’-3’) |
| --- | --- |
| TIM-F | GTTTTGGCAATCTGCCTAAGGA |
| TIM-R | GCAGCTCATACAAGGTTTCACT |
| Sp1-F | AGTTCCAGACCGTTGATGGG |
| Sp1-R | GTTTGCACCTGGTATGATCTGT |
| CERS2-F | GCTCTTCCTCATCGTTCGATAC |
| CERS2-R | CTTGCCACTGGTCAGGTAGA |
| SPTLC2-F | TGCTCACGTATGTGGGGTATG |
| SPTLC2-R | GATTGGCCGATTCCAGTTGTC |
| UGCG-F | GCTCAGTACATTGCCGAAGAT |
| UGCG-R | AGCATTCTGAAATTGGCTCACA |
| DEGS1-F | GAGATCCTGGCAAAGTATCCAGA |
| DEGS1-R | CAAACGCATAGGCCCCAAA |
| SMPD1-F | CCAGGTTACATCGCATAGTGC |
| SMPD1-R | TGATGGCGGTGAATAGACCTTT |
| ACER2-F | TGGTGCGAGGACAACTACAC |
| ACER2-R | GCAGATGGGCGGTAAAATGAA |
| ACER3-F | GGTCTGGAAAAGCGGTACATT |
| ACER3-R | GGAGTTCATCCAATAGCTGCAT |
| SGPP2-F | TCACCGCACTCCTCATCGT |
| SGPP2-R | CCGGGTTGGGCTGTAGTAATC |
| 18s-F | TGCGAGTACTCAACACCAACA |
| 18s-R | GCATATCTTCGGCCCACA |

**Supplementary Table 2 Primers used in CHIP-PCR**

| Primer Name | Sequence (5’-3’) |
| --- | --- |
| ACER2-F | ACCTGCCGGGTGCTGGGTAGTCT |
| ACER2-R | AGCGGCGAAAGCGGCTTTATGAA |
| Negative control-F | AACAGTGCAATGCTTAAAGACAA |
| Negative control-R | GAGTTGAATGAACAATGGGAGAT |

**Supplementary Table 3 Sequence of wild-type and mutated ACER2 promotor regions**

|  | Sequence |
| --- | --- |
| Wild-type | CTCGAGTTTTTATTTTAAATTTTTTTCCAGGGAGAGTTCTGGCGCTCCTT  CAATCGGGGGAGGCTGCGAGTCTGAGCCAGGAGATGGGCGTGGGCAGGCG  TGGGTAGCACCATCGAGGCCCTGGCCACCTGCCGGGTGCTGGGTAGTCTG  GAAGAGTCTGGAGCCCGGTCTCCGCCTCCAGGCGCCCCACCCTGGACCCC  TCCTCCACGCGGGTCCCGCCCCACGGTGGGCGTGGCTTCCAGGGGGAGGG  GCCGGGGAGGGTCTGCGAACGAGTAACCTCCCGCAAATATAATAAGGCGG  GGCGGAGCGCGGGAGGCCAGTTGGGAGGCGCACATCCGGCGGTTACCCGG  TGCTTCATAAAGCCGCTTTCGCCGCTGGCTGTCGCCGCGTTTTGCCTCCG  CAGCAGCTCTGGGCTCTTCTCAGCTGCGCGAGCAGCTGCTCCAATGCCCC  GGAGTGGCCATGGGCGCCCCGCACTGGTGGGACCAGCTGCAGGCTGGTAC  CATGGGGGCAGGCGTGGGTAGCACCATCGAGGCCCTGGCCACCTGCCGGG  TGCTGGGTAGTCTGGAAGAGTCTGGAGCCCGGTCTCCGCCTCCAGGCGCC  CCACCCTGGACCCCTCCTCCACGCGGGTCCCGCCCCACGGTGGGCGTGGC  TTCCAGGGGGAGGGGCCGGGGAGGGTCTGCGAACGAGTAACCTCCCGCAA  ATATAATAAGGCGGGGCGGAGCGCGGGAGGCCAGTTGGGAGGCGCACATC  CGGCGGTTACCCGGTGCTTCATAAAGCCGCTTTCGCCGCTGGCTGTCGCC  GCGTTTTGCCTCCGCAGCAGCTCTGGGCTCTTCTCAGCTGCGCGAGCAGC  TGCTCCAATGCCCCGGAGTGGCCATGGGCGCCCCGCACTGGTGGGACCAG  CTGCAGGCTGGTACCATGG |
| Mutation | CTCGAGTTTTTATTTTAAATTTTTTTCCAGGGAGAGTTCTGGCGCTCCTT  CAATCGGGGGAGGCTGCGAGTCTGAGCCAGGAGATGGGCGTGGGCAGGCG  TGGGTAGCACCATCGAGGCCCTGGCCACCTGCCGGGTGCTGGGTAGTCTG  GAAGAGTCTGGAGCCCGGTCTCCGCCTCCAGGCGCCCCACCCTGGACCCC  TCCTCCACGCGGGTCCCGCCCCACGGTGGGCGTGGCTTCCAGGGGGAGGG  GCCGGGGAGGGTCTGCGAACGAGTAACCTCCCGCAGGCGAGGCGAGGCGG  GGCGGAGCGCGGGAGGCCAGTTGGGAGGCGCACATCCGGCGGTTACCCGG  TGCTTCATAAAGCCGCTTTCGCCGCTGGCTGTCGCCGCGTTTTGCCTCCG  CAGCAGCTCTGGGCTCTTCTCAGCTGCGCGAGCAGCTGCTCCAATGCCCC  GGAGTGGCCATGGGCGCCCCGCACTGGTGGGACCAGCTGCAGGCTGGTAC  CATGGGGGCAGGCGTGGGTAGCACCATCGAGGCCCTGGCCACCTGCCGGG  TGCTGGGTAGTCTGGAAGAGTCTGGAGCCCGGTCTCCGCCTCCAGGCGCC  CCACCCTGGACCCCTCCTCCACGCGGGTCCCGCCCCACGGTGGGCGTGGC  TTCCAGGGGGAGGGGCCGGGGAGGGTCTGCGAACGAGTAACCTCCCGCAG  GCGAGGCGAGGCGGGGCGGAGCGCGGGAGGCCAGTTGGGAGGCGCACATC  CGGCGGTTACCCGGTGCTTCATAAAGCCGCTTTCGCCGCTGGCTGTCGCC  GCGTTTTGCCTCCGCAGCAGCTCTGGGCTCTTCTCAGCTGCGCGAGCAGC  TGCTCCAATGCCCCGGAGTGGCCATGGGCGCCCCGCACTGGTGGGACCAG  CTGCAGGCTGGTACCATGG |

Note: Highlight area is the mutation sequence.
