# Supplementary material for: TIMELESS regulates sphingolipid metabolism and tumor cell growth through Sp1/ACER2/S1P axis in ER-positive breast cancer
Source: Cell Death Dis. 2020 Oct 22;11(10):892. doi: 10.1038/s41419-020-03106-4 (PMC7581802; doi:10.1038/s41419-020-03106-4)
Supplement: Supplementary file 8 — Supplemental Figure legend [file 41419_2020_3106_MOESM8_ESM.docx]

**Supplementary Fig. 1**

(a) Kaplan-Meier analysis of the association between TIM and overall survival (OS), relapse free survival (RFS) and distance metastasis free survival (DMFS)of all breast cancer patients from KM plot database. (b) Kaplan-Meier analysis of the association between TIM and OS, RFS and DMFS of ER-negative breast cancer patients fromKM plot database.

**Supplementary Fig. 2**

(a-b) The interference efficiency of TIM via short hairpin (shRNA) in MCF7 and T47D cells. Values are means ± SD, ***p<0.001 (Student's t-test). (c) Morphologic characteristic of orthotopic xenograft mice transplanted with MCF7 cells treated with shNC or shTIM (n=5). (d) The interference efficiency of TIM and ACER2 of MCF7 and T47D cells in different groups ( shNC + vector, shTIM-1 + vector, shTIM-2 + vector, shTIM-1 + oeACER2， shTIM-2 + oeACER2, shNC + oeACER2-TET-on) . (e) The interference efficiency of TIM and ACER3 of MCF7 and T47D cells in different groups (shNC + vector, shTIM-1 + vector, shTIM-1 + oeACER3, shNC + oeACER3).

**Supplementary Fig. 3**

(a) Representative images of HPLC-MS in standard sample, MCF7 (oeTIM + siNC) sample, T47D (oeTIM + siNC) sample. (b) The relative activity of mitochondrial complex IV in TIM knockdown MCF7 and T47D cells and the control cells.

**Supplementary Fig. 4**

(a) Relative mRNA expression level of ACER2 of MCF7 and T47D cells in different groups (vector + siNC, oeTIM + siNC, oeTIM+siSp1-1, oeTIM + siSp1-2). (b) Cell viability of MCF7 and T47Dcells interfered by siRNA under the indicated treatments. Values are means ± SD, ***p<0.001 (Student's t-test). (c-d) Colony formation assay of MCF7 and T47D cells under the indicated treatments. Values are means ± SD, ***p<0.001 (Student's t-test). Scale bar is 5mm.

**Supplementary Fig. 5**

(a) ChIP-seq binding peak was searched using the Integrative Genomics Viewer (IGV). (b) Bioinformatics analysis was performed to verify binding sites of Sp1 within the ACER2 promoter.

**Supplementary Fig. 6**

(a) Cell viability of MCF7 and T47D cells under the indicated treatments. Values are means ± SD, ***p<0.001 (Student's t-test). (b-c) Colony formation assay of MCF7 and T47D cells under the indicated treatments. Values are means ± SD, ***p<0.001 (Student's t-test). Scale bar is 5mm.

Supplementary Table 1 Primers used for quantitative PCR in this study

Supplementary Table 2 Primers used in CHIP-PCR

Supplementary Table 3 Sequence of wild-type and mutated ACER2 promotor regions
